# Predictors of severe lupus flare: a prospective follow-up study

**DOI:** 10.1186/s41927-023-00333-y

**Published:** 2023-05-24

**Authors:** Alimohammad Fatemi, Elaheh Keivani-Boroujeni, Abbas Smiley

**Affiliations:** 1 Rheumatology Section, Department of Internal Medicine, School of Medicine, Isfahan University of Medical Sciences, Isfahan, Iran; 2 Department of Internal Medicine, School of Medicine, Isfahan University of Medical Sciences, Isfahan, Iran; 3 Westchester Medical Center, Department of Surgery, New York Medical College, 100 Woods, Valhalla, NY 10595 USA

**Keywords:** Lupus flare, Nephritis, SLEDAI, Age

## Abstract

**Background:**

Flare-up of systemic lupus erythematosus (SLE) is a common characteristic that could have deleterious effects on patients’ outcome and survival. The aim of this study was to identify the predictors of severe lupus flare.

**Methods:**

120 patients with SLE were enrolled and followed-up for 23 months. Demographic, clinical manifestations, laboratory parameters and disease activity were recorded at each visit. In addition, presence of severe lupus flare at each visit was evaluated by using the Safety of Estrogens in Lupus Erythematosus National Assessment (SELENA)-SLE disease activity index (SLEDAI) flare composite index. Predictors of severe lupus flare were obtained by backward logistic regression analyses. Predictors of SLEDAI were obtained by backward linear regression analyses.

**Results:**

During the follow-up period, 47 patients had at least one episode of severe lupus flare. Mean (SD) age of patients with severe flare versus no flare was 31.7 (7.89) and 38.3 (8.24) years, respectively (P = 0.001). Ten (62.5%) out of 16 males and 37 (35.5%) out of 104 females had severe flare (P = 0.04). History of lupus nephritis (LN) was recorded in 76.5% and 44% of patients with severe flare and no severe flare, respectively (P = 0.001). Thirty-five (29.2%) patients with high anti-double-stranded DNA (anti-ds-DNA antibody) and 12 (10%) with negative anti-ds-DNA antibody had severe lupus flare (P = 0.02). By multivariable logistic regression analysis, younger age (OR = 0.87, 95% CI 0.80–0.94, P = 0.0001), history of LN (OR = 4.66, 95% CI 1.55–14.002, P = 0.006) and high SLEDAI at the first visit (OR = 1.19, 95% CI 1.026–1.38) were the main predictors of flare. When severe lupus flare after the first visit was used as the outcome variable, similar findings were observed but, SLEDAI, although left among the final predictors in the model, was not significant. SLEDAIs in future visits were mainly predicted by Anti-ds-DNA antibody, 24-h urine protein and arthritis at the first visit.

**Conclusion:**

SLE patients with younger age, history of previous LN or high baseline SLEDAI, may need closer monitoring and follow up.

**Supplementary Information:**

The online version contains supplementary material available at 10.1186/s41927-023-00333-y.

## Background

Systemic lupus erythematosus (SLE) is a chronic systemic autoimmune disease with frequent periods of remissions and exacerbations [1]. Although its outcome has improved in the recent decades, its burden on patients and health system is still large and implementation of more effective preventive and management strategies are needed [2–4]. One of the most common characteristics of SLE is frequent disease flare that could have negative effects on the course of disease and patient’s life such as more hospitalizations, need to frequent diagnostic measures, economic burden on health system and patients, damage accrual, lower quality of life and survival [5–7]. Given numerous ominous effects of flare on patient’s outcome, the ability to timely recognize the flares as well as to identify the possible predictors are very imperative for rapid diagnosis and necessary changes in management plans.

Several studies in different populations have been done to recognize predictors of lupus flare, considering different aspects of disease such as serological and clinical features [8–13]. However, in our knowledge, no study has been conducted in our country to address this issue yet. Herein, we report a prospective study to identify the predictors of severe lupus flare by evaluating the clinical and laboratory parameters as well as the administered medications.

## Methods

### Study design

Patients who fulfilled the revised American college of rheumatology criteria for SLE [14] and visited in the university affiliated lupus clinic were studied prospectively. The regional ethics committee of medical school approved the study protocol (Code: IR.MUI.MED.REC.1398.135).

All patients signed the informed consent before enrollment.

### Data collection

In a longitudinal study, 120 patients were followed-up from June 2019 to April 2021. The intervals between visits were not scheduled in advance. In fact, the patients were visited “as needed” in routine clinical practice and according to their symptoms and health status. The medications were refilled for the next 6 months pending automatic 4–8 weekly acceptable CBC, LFT and BUN/CR and no new or exacerbation of symptoms. The patients were able to request for visit if they needed based on the new symptoms or exacerbation of the current symptoms. The doctor was also able to request to visit the patients based on lab findings. Some patients were visited every month and some were visited every six months. 744 visits were carried out. All patients were visited at least three times during the study period. 114 patients were visited 4 times, 90 patients 5 times, 75 patients 6 times, 55 patients 7 times, 31 patients 8 times, 14 patients 9 times and 5 patients were visited 10 times. Clinical manifestations as well as laboratory parameters were recorded on the first visit as the baseline and on each visit thereafter. History of nephritis was defined whether the patients had the recorded evidence of nephritis in his/her past history Disease activity was measured by SLE-disease activity index-2k (SLEDAI-2K) [15]. Damage was evaluated by Systemic Lupus International Collaborating Clinics/American College of Rheumatology damage index (SDI) [16]. Presence or absence of severe flare in each visit was evaluated by Safety of Estrogens in Lupus Erythematosus National Assessment (SELENA)-SLEDAI flare composite index [17]. In brief, severe flare was identified if one the following items was met: changes in SLEDAI > 12, new/worse manifestation of neuropsychiatric lupus, vasculitis, nephritis, myositis, platelet < 60,000, hemolytic anemia with hemoglobin < 7 mg/dl, the need to double the dosage of prednisolone or the dosage > 0.5 mg/kg/day, hospitalization for SLE, new immunosuppressive prescription or physician global assessment (PGA) > 2.5, on a scale of 0–3.

#### Exposures/predictors

Demographic characteristics as well as clinical manifestations at each visit were recorded. In addition, laboratory parameters including complete blood count, blood urea nitrogen, serum creatinine, erythrocyte sedimentation rate, C-reactive protein, anti-double-stranded DNA antibody (anti-ds-DNA antibody), serum complements, antiphospholipid antibodies were measured. Moreover, possible association between SELENA)-SLEDAI flare composite index, PGA, SDI and administered medications were assessed. The prescribed medications included prednisolone, hydroxychloroquine, mycophenolate mofetil, methotrexate, tacrolimus, cyclophosphamide, azathioprine and cyclosporine.

### Laboratory assessment

Anti-ds-DNA antibody was quantified by immunofluorescence kit (Alkides, Medipan GmbH, Germany) and the suggested cut-off value was 20 IU/ml as established by the manufacturer. Complement components, C3 and C4, were measured by turbidimetry (Aptec Diagnostics, Belgium). The corresponding normal ranges were 75–135 mg/dl and 9–36 mg/dl for C3 and C4, respectively. Anticardiolipin and anti-β2 glycoprotein I antibodies were measured by ELISA kit (Generic Assays, Germany, cut-off point: > 18 Gpl/ml) and ELISA kit (Euroimmun, Germany, cut-off point: > 24 U/ml), respectively.

### Statistical analysis

Data analyses were conducted using SPSS program (SPSS, Chicago, IL). Patients were divided into two groups: patients who had no severe lupus flare during the study period vs. those who had at least one episode of severe lupus flare. Also, patients were divided into three groups: patients who had no severe lupus flare during the study period vs. those who had one episode of severe lupus flare vs. patients with more than one episode of severe lupus flare. Categorical variables were compared between the two groups or among the three groups by chi-square test. Continuous variables were analyzed using Mann–Whitney U test and t test for nonparametric and parametric variables, respectively, to compare the differences between the two groups. Continuous variables were also compared among the three groups using ANOVA test. Univariable and multivariable backward logistic regression analyses were applied to estimate the crude and adjusted associations of different risk factors and severe lupus flare. Multivariable backward linear regression analyses were applied to estimate the adjusted associations of different risk factors and SLEDAI. Removal of 0.05 was considered for the backward elimination. P value less than 0.05 was considered significant.

## Results

120 patients with lupus were followed up in the current cohort study. They included 104 females and 16 males. The mean (SD) age of patients was 35.5 (2) years. The average (SD) disease duration was 10.5 (1.5) years. Table 1 presents the baseline categorical characteristics of patients based on having at least one episode of severe lupus flare. There were no significant differences between the two groups of severe flare vs. no severe flare in most features. But, distribution of gender, history of nephritis, active nephritis at the first visit, and positive anti-ds-DNA antibody at the first visit were significantly different between the two groups (Table 1). Different etiologies of severe lupus flare at the first visit and the cumulative frequency distribution of the etiologies of flare are presented in Table 1. It is important to remind that this is a comparison between patients with severe flare vs. those with no severe flare. The latter group included patients with moderate/mild or no flare. That’s why they may also show different etiologies of flare. Interestingly, patients with rash, arthritis, thrombocytopenia or immunologic reactions were less likely to have severe lupus flare whereas those with nephritis or neuropsychiatric symptoms were more likely to end up showing severe lupus flare (Table 1).

**Table 1:** Categorical characteristics of patients according to severe lupus flare-up vs. no severe flare

Categorical characteristics of patients		Severe lupus flare-up				*P* value
		No, N = 73 (61% of the Total)		Yes, N = 47 (39% of the Total)		
Gender, female		67	92%	37	79%	0.04
History of cardiovascular diseases		1	1.4%	0	0%	0.4
History of diabetes		0	0%	1	2%	0.4
History of nephritis		32	44%	36	76.5%	0.001
History of hypertension		18	25%	9	19%	0.5
Anticardiolipin antibody (IgG)		17	23.5%	10	21%	0.6
Anticardiolipin antibody (IgM)		13	18%	8	17%	0.75
Anti-beta 2 glycoprotein I antibody (IgM)		1	1.4%	0	0%	0.4
Anti-beta 2 glycoprotein I antibody (IgG)		6	8.5%	4	8.5%	0.9
Antiphospholipid syndrome		11	15%	5	10.5%	0.4
Severe flare at the 1^st^ visit		0	0.0%	19	40.5%	0.001
Severe flare at the 2nd visit		0	0.0%	7	15%	0.04
Severe flare at the 3rd visit		0	0.0%	6	13%	0.08
Severe flare at the 4th visit		0	0.0%	5	11%	0.15
Severe flare at the 5th visit		0	0.0%	5	11%	0.15
Severe flare at the 6th visit		0	0.0%	10	21%	0.006
Severe flare at the 7th visit		0	0.0%	5	10.5%	0.15
Severe flare at the 8th visit		0	0.0%	2	4%	0.5
Severe flare at the 9th visit		0	0.0%	3	6%	0.3
Severe flare at the 10th visit		0	0.0%	0	0%	NA
Etiology of severe flares at the first visit	Renal	3	2.50%	17	36%	0.001
	Rash	2	1.70%	0	0%	
	Immunologic	1	1.4%	0	0%	
	Arthritis	1	1.4%	0	0%	
	Renal Plus Arthritis	1	1.4%	1	2%	
	Renal Plus Thrombocytopenia	1	1.4%	0	0%	
	Renal Plus Leukopenia	0	0%	1	2%	
	Neuropsychiatric	0	0%	6	13%	
Cumulative etiologies of severe flares by the end of all visits	Renal	41	34.2%	50	42%	0.01
	Rash	8	6.7%	2	1.7%	
	Oral Ulcer	1	0.8%	0	0.0%	
	Immunologic	8	6.7%	0	0.0%	
	Leukopenia	1	0.8%	0	0.0%	
	Thrombocytopenia	4	3.3%	2	1.7%	
	Arthritis	12	10%	1	0.8%	
	Renal Plus Arthritis	1	0.8%	1	0.8%	
	Renal Plus Thrombocytopenia	1	0.8%	1	0.8%	
	Renal Plus Leukopenia	0	0.0%	1	0.0%	
	Neuropsychiatric	0	0.0%	6	5.0%	
At the first visit*:						
Anti-ds-DNA		39	53.5%	35	74.5%	0.02
Low C3		24	33%	21	44.5%	0.2
Low C4		18	25%	14	30%	0.5
Active nephritis		7	9.5%	22	47%	0.001
CRP		11	15%	9	19%	0.5
Malar rash		1	1.4%	3	6%	0.15
Discoid rash		1	1.4%	1	2%	0.9
Arthritis		2	3%	2	4%	0.65
Serositis		1	1.4%	0	0%	0.9
Use of Hydroxychloroquine		52	71%	39	83%	0.15
Use of azathioprine		9	12.5%	11	23.5%	0.1
Use of cyclophosphamide		3	4.5%	3	6%	0.7
Use of mycophenolate mofetil		18	25%	15	32%	0.4
Use of Methotrexate		2	3%	0	0%	0.5
Use of tacrolimus		9	12.5%	7	15%	0.7

*Anti-ds-DNA* anti-double stranded DNA

^*^None of the patients had any of the followings at the first visit: oral ulcer, seizure, lung involvement, heart involvement, ocular involvement, gastrointestinal involvement, or use of Cyclosporin

When the number of episodes of severe flare was taken into account, some of these differences became more prominent (Additional file 1: Table S1). For instance, 7% of 73 patients with no severe lupus flare, 23% of 35 patients with one episode of severe flare and 25% of 12 patients with more than one episode of severe flare were male (P = 0.03). Also, 44% of patients with no severe flare, 74.5% of patients with one episode of severe flare and 83.5% of patients with more than one episode of severe flare had history of nephritis (P = 0.002). Interestingly, almost the same distribution was observed in terms of anti-dsDNA antibody at the first visit: 52% vs. 74.5% vs. 83.5%, respectively (P = 0.02).

Table 2 shows the continuous variables based on having at least one episode of severe lupus flare. There were no significant differences between the two groups of severe flare vs. no severe flare in most features. Patients who had at least one severe flare were about 7 years younger than those with no severe flare (Table 2). Patients with severe flare had significantly higher levels of 24-h urine protein at the first visit, higher scores of SLEDAI at the first visit and higher cumulative dose of prednisolone than those with no severe lupus flare (Table 2). The same variables were significantly different among the three groups of no severe flare vs. those with one episode of severe flare vs. patients with more than one episode of severe lupus flare. Their mean age was 38 vs. 32 vs. 30.8 years, respectively (P < 0.001). The SLEDAI was 2.9 vs. 6.2 vs. 6.6, respectively (P < 0.001). The cumulative dose of prednisolone was 2,186 vs. 4,819 vs. 5,116 mg, respectively (P < 0.001).

**Table 2:** Continuous variables in lupus patients according to having severe lupus flare-up vs. no severe flare

Continuous characteristics of patients	Severe Lupus Flare-up				*p*
	No, N = 73		Yes, N = 47		
	Mean	SD	Mean	SD	
Age, years	38.30	8.24	31.70	7.89	0.001
Disease duration, years	11.23	7.46	9.04	7.15	0.10
Duration of follow-up, months	20.97	2.94	19.57	3.85	0.03
24-urine protein at the first visit, mg/dl	260.83	491.86	763.80	1011.56	0.0001
Creatinine at the first visit	0.90	0.27	0.93	0.20	0.6
BUN at the first visit	14.10	7.56	15.07	9.51	0.6
GFR at the first visit	88.97	22.06	92.50	23.40	0.4
ESR at the first visit	19.52	17.34	19.31	13.26	0.90
WBC at the first visit	5665	2248	6242	2382	0.2
Platelets at the first visit	221,411	73,905	239,893	75,201	0.2
Hemoglobin at the first visit	12.29	1.55	12.68	2.16	0.25
PGA at the first visit	0.29	0.68	1.12	1.29	0.0001
SLEDAI at the first visit	2.75	2.87	6.53	4.92	0.0001
Prednisolone dose at the first visit, mg/d	5.17	6.50	6.27	7.87	0.40
Cumulative dose of prednisolone, mg	2201	2332	4816	2870	0.0001

BUN, blood urea nitrogen; GFR, glomerular filtration rate; ESR, erythrocyte sedimentation rate; PGA, physician global assessment; SLEDAI, systemic lupus erythematosus activity index

Univariable and multivariable associations of different patient characteristics and severe lupus flare are presented in Table 3. Univariable evaluations demonstrated significant associations of severe lupus flare and the following variables: age, sex, anti-dsDNA antibody at the first visit, history of nephritis and SLEDAI. After controlling for all confounders in multivariable logistic regression model with backward stepwise process, the following significant predictors of severe lupus flare were left in the final model: age, history of nephritis and SLEDAI (Table 3). Every one-year older age decreased the odds of severe lupus flare by about 13%. One score increase in SLEDAI increased the odds of severe lupus flare by 19%. Finally, history of nephritis was the most powerful risk factor of the future attack of severe lupus flare which increased the odds by more than 4.5 times (Table 3). It is important to mention that PGA was not considered in the regression model because it was strongly correlated with SLEDAI. In fact, the coefficient correlation of PGA and SLEDAI is 0.81 (P < 0.0001). Then, adding PGA to the regression model would cause multi-collinearity issue.

**Table 3:** Univariable and multivariable logistic regression analyses with backward elimination process to find the predictors of severe lupus flare-up

Independent variables	OR	95% confidence interval (CI)		*P*	OR	95% confidence interval (CI)		*P*
		Lower	Upper			Lower	Upper	
Age	0.903	0.857	0.952	0.0001	0.869	0.804	0.939	0.0001
History of nephritis	4.193	1.85	9.505	0.001	4.66	1.551	14.002	0.006
SLEDAI at the first visit	1.288	1.144	1.449	0.0001	1.191	1.026	1.382	0.02
Sex	3.018	1.016	8.966	0.047	Removed by backward elimination process			
Disease duration	0.958	0.909	1.011	0.10				
History of cardiovascular diseases	1	1	1	0.99				
History of diabetes	1	1	1	0.99				
History of hypertension	0.724	0.294	1.781	0.50				
Anticardiolipin antibody (IgG)	0.801	0.328	1.953	0.65				
Anticardiolipin antibody (IgM)	0.858	0.324	2.274	0.75				
Anti-beta 2 glycoprotein I antibody (IgM)	1	1	1	0.99				
Anti-beta 2 glycoprotein I antibody (IgG)	0.968	0.257	3.642	0.95				
Antiphospholipid syndrome	0.621	0.2	1.924	0.40				
Anti-ds-DNA at the first visit	2.543	1.142	5.664	0.02				
Low C3 at the first visit	1.649	0.776	3.506	0.20				
Low C4 at the first visit	1.296	0.57	2.946	0.50				
Creatinine at the first visit	1.502	0.345	6.528	0.60				
24-hour proteinuria at the first visit	1.006	0.989	1.023	0.50				
BUN at the first visit	1.014	0.97	1.059	0.55				
GFR at the first visit	1.007	0.99	1.024	0.40				
ESR at the first visit	0.999	0.975	1.024	0.95				
CRP at the first visit	1.403	0.529	3.717	0.50				
WBC at the first visit	1	1	1	0.20				
Platelet at the first visit	1	1	1	0.20				
Hemoglobin at the first visit	1.127	0.916	1.386	0.25				
Dose of prednisolone at the first visit	1.022	0.97	1.077	0.40				
Cumulative dose of prednisolone	1.000	1.000	1.001	0.001				
Use of hydroxychloroquine at the first visit	1.969	0.789	4.911	0.15				
Use of azathioprine at the first visit	2.173	0.823	5.737	0.10				
Use of cyclophosphamide at the first visit	1.591	0.307	8.235	0.60				
Use of mycophenolate mofetil at the first visit	1.432	0.636	3.226	0.40				
Use of methotrexate at the first visit	1	1	1	0.99				
Use of tacrolimus at the first visit	1.244	0.43	3.606	0.70				

SLEDAI, systemic lupus erythematosus activity index; Anti-ds-DNA, Anti-double stranded DNA, BUN, blood urea nitrogen; GFR, glomerular filtration rate; ESR, erythrocyte sedimentation rate; CRP, C-reactive protein

When patients with severe flare at the first visit were excluded and severe flare after the first visit was used as the outcome variable, the repeat regression model showed almost similar findings to those of the Table 3 except one difference; SLEDAI lost its significance, although it left among the final predictors in the model. This could be mainly due to the reduced sample size (Additional file 2: Table S2).

**Fig. 1:**
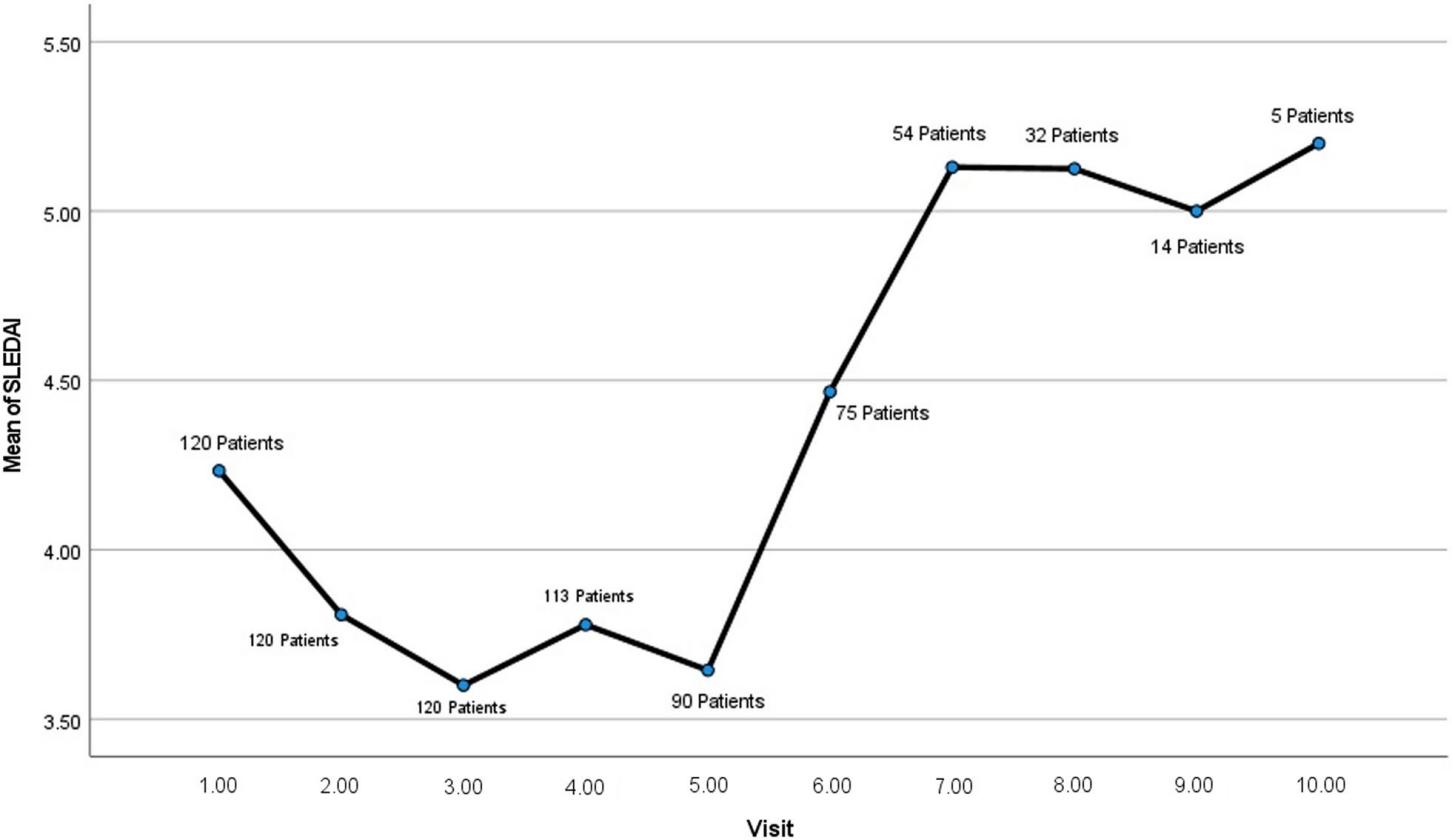
Mean SLEDAI in different visits along with the number of patients within each visit

On the other hand, multivariable linear regression analyses with backward elimination process were carried out to find the predictors of SLEDAI in the next visits according to the potential risk factors recorded at the first visit (Table 4). It demonstrated that SLEDAIs in future visits were mainly predicted by Anti-ds-DNA antibody, 24-h urine protein and arthritis at the first visit. The descriptive characteristics of SLEDAI during different visits are presented in Table 5. The mean SLEDAI in different visits are also plotted in Fig. 1. Since the less stable patients were visited more often, the average SLEDAI after the fifth visit increased significantly (Fig. 1).

**Table 4:** Multivariable linear regression model to assess the predictors of SLEDAI according to variables recorded at the first visit

Independent variables at the first visit	SLEDAI														
	2nd visit			3rd visit			4th visit			5th visit			6th visit		
	*B*	95% CI		*B*	95% CI		*B*	95% CI		*B*	95% CI		*Β*	95% CI	
Anti-ds-DNA*	1.28	0.24	2.32	1.47	0.45	2.48	2.15	1.09	3.21				3.07	1.07	5.07
Low C3*	2.93	1.65	4.22												
Low C4*	− 1.56	− 2.88	− 0.24												
24-hour urine protein*	0.002	0.001	0.003	0.002	0.001	0.003	0.002	0.001	0.003	0.002	0.001	0.003			
Arthritis*	4.83	2.21	7.44	3.47	0.91	6.04				3.84	0.96	6.73			
Other Independent Variables from Table 3	Removed via backward elimination			Removed via backward elimination			Removed via backward elimination			Removed via backward elimination			Removed via backward elimination		

*Anti-ds-DNA* anti-double stranded DNA

*P < 0.05

**Table 5:** SLEDAI descriptions during different visits

Visit number	# of patients	SLEDAI					
		Mean	SD	95% CI		Min	Max
				Lower	Upper		
1	120	4.23	4.21	3.47	4.99	0	18
2	120	3.80	3.66	3.14	4.47	0	16
3	120	3.60	3.42	2.98	4.21	0	16
4	114	3.77	3.87	3.05	4.50	0	16
5	90	3.64	3.28	2.95	4.33	0	16
6	75	4.46	4.13	3.51	5.41	0	20
7	55	5.12	4.06	4.02	6.23	0	16
8	31	5.12	4.19	3.61	6.63	0	16
9	14	5.00	3.11	3.20	6.79	2	12
10	5	5.20	4.14	0.05	10.34	2	12
Total	744	4.07	3.81	3.79	4.34	0	20

*SLEDAI* systemic lupus erythematosus activity index

## Discussion

In the current study, we investigated the possible effects of multiple clinical and laboratory parameters on subsequent lupus flare. It was not surprising that male patients experienced more flares than female patients. More severe course of lupus in males have been demonstrated in previous studies [18, 19]. However, this association was not persisted in the final model when other covariates were considered. Patients with higher levels of anti-ds-DNA antibody at the first visit had higher SLEDAI and more severe flare in their follow-up. Though, it was not a predictive factor of flare in logistic regression analysis, it was a predictive factor of SLEDAI in linear regression analysis. The association between changes of anti-ds-DNA antibody and SLE exacerbation was shown in many studies [13, 20–22], although some others failed to observe it [23–25]. This inhomogeneity among various studies might be due to different cut-off points of high anti-ds-DNA antibody that led to different sensitivity and specificity of the test. Another explanation could be different times between the rise in anti-ds-DNA antibody and the subsequent flare [11]. The association between antiphospholipid antibodies and lupus flare was not shown in previous studies [26, 27]. Our results also didn’t confirm this association in multivariable regression analysis.

In our study, we did not find the disease duration as a predictor of severe flare. Consistent with our observation, some previous studies showed the same [9, 10, 13]. However, other researches such as those have done by Conti et al. [28] and Cho et al. [29] demonstrated longer disease duration as a harbinger of flare. The authors proposed that this might be due to higher patients’ adherence to immunosuppressive medications in the early disease or owing to long-standing immune activation resulting in higher disease activity.

In our study, younger age was one of the predictors of severe flares. Previous studies showed inconsistent associated findings. Some demonstrated that the younger aged patients were more prone to flares than the older ones which could be explained by the higher prevalence of LN [9].

The current study showed no association between corticosteroid or other immunosuppressants and lupus flare in the regression analysis. Previous studies demonstrated conflicting results. In Minowa et al. [8] and Petri et al. [13] studies, corticosteroid at baseline was not associated with subsequent flare in multivariable analysis. In addition, baseline immunosuppressant medications were not the predictors of flare [13]. On the other hand, in some investigations such as Ineˆs et al. study [9], baseline immunosuppressive medications were identified as the predictor of SLE flare that might be due to more severity of disease at the baseline.

Although efficacy of antimalarial drugs is a well-known concept in reducing the frequency of flares, lowering the mortality rate and improving the survival [30–32], the current study didn’t show its preventing effects on flares. Conflicting results were raised by previous studies regarding the inhibitory effects of these medications on flare. For instance, hydroxychloroquine was associated with lower flare rates in Canadian studies [33, 34], although this picture was not seen in Petri et al. study [13]. It should be noted that the former studies enrolled the patients who were in remission clinically, but the latter included those with active disease, an issue that might explain the different results.

In our study, in line with other studies [13, 35], higher SELDAI at the first visit was one of the main predictors of flare. Among all potential predictors explored by backward multivariable regression analysis, history of LN was the most powerful one. In previous studies, LN [9, 13], thrombocytopenia [8], neuropsychiatric lupus [13, 28], anemia and lymphopenia [23] were among the main predictors of lupus flare in different studies. These differences might be due to different populations, different study designs, and various durations of follow-up.

We also investigated the association between clinical and laboratory parameters in the first visits and high SLEDAI in the follow-up visits. Hypocomplementemia in the first visit was not a predictor of SLEDAI in the subsequent visits. Although most previous studies reported the negative correlation between the serum complement levels and the disease activity [29, 36–38], a few ones did not show it [39].

Consistent with other studies, anti-ds-DNA in the first visit was able to predict SLEDAI in the future [13, 36, 37]. Proteinuria in the first visit is another predictor of SLEDAI in the future. It is not surprising as proteinuria is known as a biological maker of disease activity [40]. In line with previous studies, arthritis was found as a predictor of higher SLEDAI in the next visits [38, 41, 42].

In our cohort, 39% of patients experienced severe flares during follow-up. The severe flare rates in some other studies were as follows: 7% in Italy [28], 17% in Portugal [9], 23–32% in a multicenter-multinational study [13], 35% in Canada [10], 38% in Norway [43], 47% in Italy [26], 53–71% in USA [21, 23, 44] and 66% in Germany [23]. In addition to the above-mentioned clinical and laboratory parameters which are considered as the potential predictors of lupus flare, other social, habitual and environmental issues might be contributing to the different disease activities and flare rates across the countries. For instance, non-Caucasian ethnicity such as Black African descent has been reported as a poor prognostic indicator of disease outcome [12]. However, in another large research on 1846 lupus patients in 9 countries from Asia–Pacific region, no association between ethnicity and disease activity was found [45].

The economic indices such as social wealth also proposed as a predictor of disease activity which should take into consideration in developing countries [45]. Environmental factors such as air pollution and climate changes also have been addressed as the associative factors on flare patterns, an important issue which should be bear in mind when comparing prevalence of lupus flares in different regions. For instance, Stojan et al. showed that hematologic and renal flares were associated positively and negatively with climate temperature, respectively [46].

Finally, the beneficial effects of healthy life style on patients with SLE can’t be overlooked. It has been demonstrated that physical inactivity is more common in lupus patients than in the general population [47]. On the other hand, obesity is independently associated with lupus activity and newly developed LN [48]. Unfortunately, the prevalence of inactivity in adult population in our country can be as high as 70% [49] which might explain partly the higher disease activity and flare rate in our patients.

The main strengths of the current study were its prospective design and the relatively long-term follow up with no scheduled visits in advance. In fact, scheduling the next visit of patients was PRN. It means we didn’t set a specific date for the next visit in advance. We believe this was more compatible with the real patients’ life pattern since they mostly seek medical attention when needed, not on a regular basis, but it would be more rational for research purposes if there were a pre-specified time-line protocol for follow-up of the patients. Our study had some limitations. Considering other autoantibodies such as anti-C1q or biological markers like chemokines and cytokines could draw a more precise picture and better understanding of possible predictors of flare. In addition, this was a single center study. The patients with more severe disease are more frequently referred to the academic clinics, an issue that could impede the generalizability of the results.

## Conclusion

In summary, a previous history of LN, younger age and higher SLEDAI were independent predictors for severe SLE flare. Larger and longer and multicenter follow-up studies could achieve a better understanding of the predictors of severe lupus flare.

## Supplementary Information

**Additional file 1.** Categorical characteristics of patients according to more than one severe lupus flare-up vs. one severe flare vs. no severe flare.

**Additional file 2.** Multivariable logistic regression analysis with backward elimination process to find the predictors of severe lupus flare when considering severe lupus flares after the first visit as the outcome variable. SLEDAI: systemic lupus erythematosus activity index.

## Data Availability

The datasets generated and/or analyzed during the current study are not publicly available due their containing information that could compromise the privacy of research participants, but are available from the corresponding author on reasonable request.

## Abbreviations

anti-ds-DNA antibody: Anti-double-stranded DNA antibody
LN: Lupus Nephritis
PGA: Physician Global Assessment
SLE: Systemic Lupus Erythematosus
SLEDAI-2K: Systemic Lupus Erythematosus-disease activity index-2k
SDI: Systemic Lupus International Collaborating Clinics/American College of Rheumatology damage index
SELENA: Safety of Estrogens in Lupus Erythematosus National Assessment
