# Supplementary material for: Predictors of severe lupus flare: a prospective follow-up study
Source: BMC Rheumatol. 2023 May 24;7:10. doi: 10.1186/s41927-023-00333-y (PMC10207823; doi:10.1186/s41927-023-00333-y)
Supplement: Supplementary file 1 — Additional file 1. Categorical characteristics of patients according to more than one severe lupus flare-up vs. one severe flare vs. no severe flare. [file 41927_2023_333_MOESM1_ESM.docx]

| **Categorical Characteristics of Patients** | **Severe Lupus Flare-up** | | | | | | **P**  **value** |
| --- | --- | --- | --- | --- | --- | --- | --- |
|  | **No**  **N=73 (61%)** | | **One Flare**  **N=35 (29%)** | | **>One Flare**  **N=12 (10%)** | |  |
| Gender, Female | 68 | 93% | 27 | 77% | 9 | 75% | 0.03 |
| History of Cardiovascular Diseases | 1 | 1.5% | 0 | 0% | 0 | 0% | 0.7 |
| History of Diabetes | 0 | 0% | 1 | 3% | 0 | 0% | 0.3 |
| History of Nephritis | 32 | 44% | 26 | 74.5% | 10 | 83.5% | 0.002 |
| History of Hypertension | 18 | 25% | 8 | 23% | 1 | 8.5% | 0.5 |
| Anticardiolipin Antibody (IgG) | 18 | 25% | 4 | 12% | 5 | 42% | 0.07 |
| Anticardiolipin Antibody (IgM) | 14 | 19% | 5 | 15% | 2 | 17% | 0.7 |
| Anti-beta 2 Glycoprotein I Antibody (IgM) | 1 | 1.5% | 0 | 0% | 0 | 0% | 0.7 |
| Anti-beta 2 Glycoprotein I Antibody (IgG) | 6 | 9% | 2 | 6% | 2 | 17% | 0.5 |
| Antiphospholipid Syndrome | 11 | 15% | 3 | 9% | 2 | 17% | 0.6 |
| Severe Flare at the 1^st^ Visit | 0 | 0% | 14 | 40% | 5 | 42% | 0.0001 |
| Severe Flare at the 2^nd^ Visit | 0 | 0% | 5 | 14.5% | 2 | 17% | 0.009 |
| Severe Flare at the 3^rd^ Visit | 0 | 0% | 3 | 9% | 3 | 25% | 0.004 |
| Severe Flare at the 4^th^ Visit | 0 | 0% | 3 | 9% | 2 | 17% | 0.045 |
| Severe Flare at the 5^th^ Visit | 0 | 0% | 2 | 6% | 2 | 17% | 0.4 |
| Severe Flare at the 6^th^ Visit | 0 | 0% | 4 | 12% | 6 | 50% | 0.0001 |
| Severe Flare at the 7^th^ Visit | 0 | 0% | 3 | 9% | 2 | 17% | 0.1 |
| Severe Flare at the 8^th^ Visit | 0 | 0% | 0 | 0% | 2 | 17% | 0.004 |
| Severe Flare at the 9^th^ Visit | 0 | 0% | 1 | 3% | 2 | 17% | 0.5 |
| Severe Flare at the 10^th^ Visit | 0 | 0% | 0 | % | 0 | 0% | NA |
| At the First Visit*: |  | | | | | |  |
| Anti-ds-DNA | 38 | 52% | 26 | 74.5% | 10 | 83.5% | 0.02 |
| Low C3 at the First Visit | 24 | 33% | 16 | 46% | 5 | 41.5% | 0.4 |
| Low C4 at the First Visit | 19 | 26% | 10 | 28.5% | 3 | 24.5% | 0.9 |
| Active Nephritis at the First Visit | 7 | 9.5% | 17 | 48.5% | 5 | 41.5% | 0.001 |
| CRP at the First Visit | 11 | 15% | 6 | 17% | 3 | 25% | 0.7 |
| Malar Rash at the First Visit | 1 | 1.5% | 1 | 3% | 2 | 17% | 0.02 |
| Discoid Rash at the First Visit | 1 | 1.5% | 0 | 0% | 1 | 8.5% | 0.1 |
| Arthritis at the First Visit | 2 | 3% | 1 | 3% | 1 | 8.5% | 0.6 |
| Serositis at the First Visit | 1 | 1.5% | 0 | 0% | 0 | 0% | 0.7 |
| Use of Hydroxychloroquine at the First Visit | 51 | 71% | 29 | 83% | 11 | 91.5% | 0.1 |
| Use of Azathioprine at the First Visit | 10 | 14% | 9 | 26% | 1 | 8.5% | 0.2 |
| Use of Cyclophosphamide at the First Visit | 3 | 4.5% | 3 | 9% | 0 | 8% | 0.4 |
| Use of mycophenolate mofetil at the First Visit | 17 | 23.5% | 11 | 31.5% | 5 | 41.5% | 0.3 |
| Use of Methotrexate at the First Visit | 2 | 3% | 0 | 4% | 0 | 0% | 0.5 |
| Use of Tacrolimus at the First Visit | 8 | 11% | 4 | 12% | 4 | 34% | 0.1 |

*Anti-ds-DNA*, Anti-double stranded DNA

*None of the patients had any of the followings at the first visit: oral ulcer, seizure, lung involvement, heart involvement, ocular involvement, gastrointestinal involvement, or use of Cyclosporin.
