# Supplementary material for: Predictors of severe lupus flare: a prospective follow-up study
Source: BMC Rheumatol. 2023 May 24;7:10. doi: 10.1186/s41927-023-00333-y (PMC10207823; doi:10.1186/s41927-023-00333-y)
Supplement: Supplementary file 2 — Additional file 2. Multivariable logistic regression analysis with backward elimination process to find the predictors of severe lupus flare when considering severe lupus flares after the first visit as the outcome variable. SLEDAI: systemic lupus erythematosus activity index. [file 41927_2023_333_MOESM2_ESM.docx]

| **Independent Variables** | **OR** | **95% Confidence Interval**  **Lower Upper** | | | **P** |
| --- | --- | --- | --- | --- | --- |
| **Age** | 0.89 | 0.82 | 0.96 | | 0.002 |
| **Sex** | 3.57 | 0.81 | 15.75 | | 0.09 |
| **History of Nephritis** | 4.02 | 1.35 | 12.00 | | 0.01 |
| **SLEDAI in first visit** | 1.16 | 0.99 | 1.36 | | 0.07 |
| **All Other**  **Independent Variables**  **Presented in Table 3** | | | | Removed By Backward Elimination | |

Supplementary Table 2. Multivariable logistic regression analysis with backward elimination process to find the predictors of severe lupus flare when considering severe lupus flares after the first visit as the outcome variable. SLEDAI: systemic lupus erythematosus activity index.
